# Controlling and Predicting the Dissolution Kinetics of Thermally Oxidised Mesoporous Silicon Particles: Towards Improved Drug Delivery

**DOI:** 10.3390/pharmaceutics11120634

**Published:** 2019-11-28

**Authors:** Feng Wang, Timothy J. Barnes, Clive A. Prestidge

**Affiliations:** 1 School of Pharmacy and Medical Sciences, University of South Australia, Adelaide, SA 5000, Australia; frank.wang@monash.edu (F.W.); tim.barnes@unisa.edu.au (T.J.B.); 2 ARC Centre of Excellence in Convergent Bio-Nano Science and Technology, University of South Australia, Adelaide, SA 5000, Australia

**Keywords:** porous silicon, drug delivery system, dissolution, oxidation, Fourier transform infrared (FTIR), kinetics

## Abstract

Porous silicon (pSi) continues to receive considerable interest for use in applications ranging from sensors, biological scaffolds, therapeutic delivery systems to theranostics. Critical to all of these applications is pSi degradation and stabilization in biological media. Here we report on progress towards the development of a mechanistic understanding for the dissolution behavior of native (unoxidized) and thermally oxidized (200–600 °C) pSi microparticles. Fourier transform infrared (FTIR) spectroscopy was used to characterize the pSi surface chemistry after thermal oxidation. PSi dissolution was assessed using a USP method II apparatus by monitoring the production of orthosilicic acid, and the influence of gastro-intestinal (GI) fluids were examined. Fitting pSi dissolution kinetics with a sum of the exponential model demonstrated that the dissolution process strongly correlates with the three surface hydride species and their relative reactivity, and was supported by the observed FTIR spectral changes of pSi during dissolution. Finally, the presence of GI proteins was shown to hamper pSi dissolution by adsorption to the pSi surface acting as a barrier preventing water attack. These findings are significant in the optimal design of pSi particles for oral delivery and other controlled drug delivery applications.

## 1. Introduction

More than 20 years after the initial discovery of porous silicon (pSi) photoluminescence by Professor Canham, and his subsequent work on the fabrication of bioactive pSi [1], there remains significant ongoing interest in its biomedical applications, including therapeutic delivery, medical imaging, and theranostics [2,3,4,5,6,7,8,9,10,11,12,13]. PSi possesses a highly tuneable porous nanostructured network and can be fabricated into a variety of different forms, ranging from thin films [14], nanowires [15], and micro- and nanoparticles [6,16,17,18]. PSi is typically produced via an electrochemical etching process (e.g., see [19]); however, there are ongoing efforts to more efficiently produce pSi nanoparticles (e.g., [20]). The surface chemistry of pSi also offers opportunities for extensive modification, e.g., thermal oxidation [21,22,23], amination [24], and chemical modification [19,25,26].

Many biomedical applications of pSi are influenced and dependent on the balance between dissolution/erosion versus oxidation of the silicon matrix that occurs in aqueous bio-relevant environments. The Si–Si and Si–H bonds are intrinsically weaker than their oxidized analogues, Si–O–Si and Si–OH [27]. When native pSi is submerged in water, the initial reaction occurring is the oxidation of the Si–H surface species by solvated OH, which subsequently acts as a nucleation substitute for removal of the oxidized surface hydrides to form orthosilic acid, as described in Equations (1) and (2). Once the surface Si–H has been removed a new layer of surface Si–H is formed, and the pSi erosion cycle continues [28,29,30].

≡Si – SiH(s) + HOH → ≡Si – SiOH(s) + H_2_(g) (1)

≡Si – SiOH(s) + HOH → ≡Si – H(s) + Si(OH)_4_(aq) (2)

Previous pSi dissolution studies have typically involved direct visualization/monitoring of a planar pSi wafer surface using techniques such as atomic force microscope (AFM), scanning electron microscope (SEM) [1,31,32], photoluminescent and infrared spectroscopies [33,34]. However, these approaches provide only qualitative or semi-quantitative topographic and/or pSi speciation information to assess pSi degradation. Alternatively, orthosilicic acid, the soluble product of pSi degradation, can be quantitatively measured using either a molybdenum blue assay [35] or inductively coupled plasma atomic emission spectroscopy (ICP-AES), and hence accurate mass loss kinetics of pSi during dissolution can be obtained. Using this approach, Anderson et al. investigated the dissolution of pSi films with varied porosity (60–80%) and over a pH range 2 to 8; however, they did not elucidate specific dissolution mechanisms in their analysis [36]. A number of studies have demonstrated correlations between drug release kinetics and pSi degradation as well as the influence of the local tissue environment [3,37,38,39].

We have previously reported on the use of thermally oxidized pSi microparticles for oral delivery of poorly water-soluble non-steroidal anti-inflammatory drugs [5,40]. In this study, we quantitatively investigate the dissolution kinetics of both native (unoxidised) and thermally oxidized (200 to 600 °C) pSi microparticles in buffer solutions and simulated gastro-intestinal (GI) fluids, and use Fourier transform infrared (FTIR) spectroscopy to determine surface chemical changes. The pSi dissolution kinetics was fitted with a sum of exponentials mathematical model, which reflects the pSi surface speciation. These findings give improved insight into pSi particle dissolution with specific reference to its use in oral delivery.

## 2. Materials and Methods

### 2.1. Materials

PSi powder and wafer samples (p-type silicon) used in this investigation were supplied by pSiMedica Ltd. (Malvern, UK) and showed a porosity of ~70%, a specific surface area (SSA) of 310.2 m^2^/g, and an average particle size of 49.4 µm (note: D_10_ 23.2, D_90_ 130.4 µm) as determined by a laser diffraction (Malvern Mastersizer 2000 software, version 4.0, Malvern Mastersizer 2000, Malvern Co, Malvern, UK). PSi thermal oxidation was performed in a clean oven by heating in air at 40 K min^−1^ to a specific oxidation temperature in the range 473–873 K for 1 h, before convective cooling to ambient temperature.

Tween 80 (Polysorbate 80) and sodium dodecyl sulfate (SDS) were purchased from Chem-Supply Pty Ltd., Adelaide, Australia. Trizma^®^ buffer solution pH 7.2 (1 M, tris(hydroxymethyl)aminomethane HCl), pancreatin from porcine pancreas (activity equivalent to 8× USP specification) and pepsin (lyophilized powder from porcine gastric mucosa, 800–2,500 units/mg protein), were purchased from Sigma-Aldrich Australia Pty Ltd, Sydney, Australia. All the other chemicals were analytical grade used as received. High purity water (Milli-Q^®^, <18 mΩ cm^−1^) was used throughout the experiments.

### 2.2. In Vitro pSi Dissolution

pSi dissolution was conducted with a VanKel™ (VK 6010) dissolution test station attached with apparatus USP II (paddles, Edison, NJ, USA) at 37.0 °C (± 0.5). PSi particles (~50 mg, accurately weighed) were pre-wetted with 200 µL of ethanol and then transferred into 500 mL of 0.05 M Tris buffer solution at pH 7.2 (or alternative dissolution medium as indicated). At predetermined time points, aliquots of media (5 mL) were collected and filtered with a Millex^®^ syringe filter (0.22 µm PTFE membrane, Merck, Darmstadt, Germany). The aliquots were analyzed to determine the concentration of silicic acid using a validated molybdenum blue spectroscopic assay (Varian Cary® 50 UV-Vis Spectrophotometer, Walnut Creek, CA, USA). For dissolution experiments performed in phosphate buffer solution (pH 6.8), the samples were analyzed using ICP-AES (Horiba Jobin-Yvon ACTIVA ICP-AES spectrometer, Longjumeau, France), since phosphate interferes with the molybdenum blue assay. All the experiments were conducted in duplicate unless otherwise indicated.

### 2.3. Fourier Transform Infrared (FTIR) Spectroscopy

Infrared transmission spectra of pSi particle samples were obtained with a Nicolet Magna-IR 750™ FTIR spectrometer equipped with a liquid nitrogen-cooled mercury-cadmium-telluride (MCT) detector (Madison, WI, USA). The particles were pressed into KBr discs and 256 scan spectra were recorded with a 4 cm^−1^ resolution. Additionally, solid residues from the dissolution study were extracted, thoroughly rinsed with Milli-Q water and then dried at 105 °C for 2 h in an oven prior to FTIR analysis.

### 2.4. Contact Angle Measurements

Water contact angle measurements were performed on pSi wafers using a static sessile drop method (Optical Contact Angle system, OCA15EC, SCA 20 version 2 software). The wafers were either unoxidized or thermally oxidized using equivalent conditions to those used for the pSi microparticles.

## 3. Results

### 3.1. Preparation and Characterisation of Thermally Oxidised pSi Microparticles

Thermal oxidation of pSi particles was carried out at 200, 300, 400, 500, and 600 °C for 1 h in a clean furnace, open to the atmosphere. The surface area and porous nature of the thermally oxidized pSi samples are presented in Table 1.

**Figure 1:**
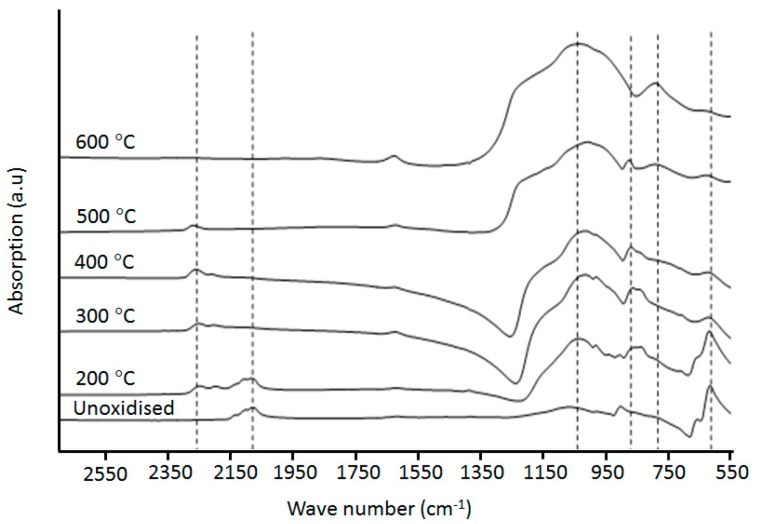
Infrared spectra of pSi particles treated at different temperatures.

Upon thermal oxidation of the pSi particles, there is a reduction in the surface area and mesopore volume; this is due to the increased molecular volume of silicon oxidation products, causing constriction of the mesoporous pathways within the pSi. The changing Si speciation upon thermal oxidation was monitored using FTIR spectroscopy, with the IR spectra for both the native and thermally oxidized pSi shown in Figure 1. For the native, unoxidized pSi, a composite band with triplet peaks at 2140, 2105, and 2075 cm^−1^ was observed. These absorption bands were assigned to Si-Hx stretching modes (*x* = 3, 1, 2; from high frequency to low) [41,42], the higher intensity of the peak at 2075 cm^−1^ indicated that dihydride (SiH_2_) was the more predominant surface species, in agreement with previous reports [43,44]. Upon thermal oxidation of the pSi at 200 °C, this band was observed to shift to a higher frequency, which according to previous literature is attributed to pore propagation occurring only in the (100) direction [45,46].

In the finger print region (<1000 cm^−1^), a sharp absorption band at 615 cm^−1^ with a shoulder at 660 cm^−1^ was observed for native and 200 °C oxidized pSi samples, attributed to Si–Si crystal mode and Si–H_2_ wagging modes, respectively [27,47], indicating that only the surface hydride species underwent oxidation, rather than the bulk Si. A new band at 860 cm^−1^ was observed with increasing oxidation temperature of the pSi, which was assigned as an oxidised O–SiHx deformation mode [48]. The sharp band at 905 cm^−1^ was attributed to a Si–H_2_ scissoring mode, while the broad band at 1030 cm^-1^ was attributed to Si–O–Si asymmetric bending. The increased intensity of the 1030 cm^−1^ band and the decreased intensity of absorption (Si–H_2_ scissoring) implies that mild oxidation had occurred. This feature became more pronounced with increasing oxidation temperature (>300 °C). The presence of this band (1030 cm^−1^) in samples not exposed to high temperatures indicates that oxidation of the pSi sample was slowly occurring under ambient conditions. Finally, a band was observed at 2250 cm^−1^, associated with the backbond oxidized Si–Hx, i.e., (Si–O–)Si–Hx [42].

The spectra of the thermally oxidized pSi particles at 300 °C and above displayed more extensive evidence of oxidation characteristics. The bands at 660, 905, and 2075–2140 cm^−1^ disappeared; these bands were associated with Si–H_2_ wagging, scissoring, and Si–Hx stretching modes. The band at 2250 and 2205 cm^−1^ due to the backbond oxidized Si–Hx stretching became more profound. These absorptions could be precisely assigned to OxSiHx groups with oxygen-saturated state, i.e., O_3_SiH (2250 cm^−1^), O_2_SiH_2_ (2200 cm^−1^), OSiH_3_ (2160 cm^−1^) according to the literature [27]. A broad band peaking at 1030 cm^−1^ became more intense with a growing protruded shoulder compared to that at 200 °C, in agreement with previous observation [49]. The band at 612 cm^−1^ associated with bulk Si–Si decreased, indicating oxidation had started altering the Si–Si “skeleton” at 300 °C, something that has been referred to previously as a threshold temperature for silicon thermal oxidation [43,47]. The infrared spectrum obtained for OXpSi-400 was similar to that for OXpSi-300, except that the band at 2250 cm^−1^ had increased in intensity. 

The pSi samples treated at 500–600 °C showed more significant oxidation features in their infrared spectra. For oxidation at >500 °C, a new absorption band appeared at 800–790 cm^−1^ due to the asymmetric and symmetric mode of Si–O, which is often observed with fully oxidized silicon; its intensity further increased with oxidation at 600 and 800 °C (data not shown). The single band at 2250 cm^−1^ instead of the duplet and triplet peaks from pSi sample oxidized at 300–400 °C indicated that there was only SiH with fully oxidized Si–Si backbonds (i.e., Si–O–Si) on pSi surfaces. The phenomenon of Si–Si backbond oxidation can be explained by bond energy calculations, i.e., the bond strength of surface hydride (Si–H) increases with the increase of oxidation degree of its Si–Si backbond. Thus, the survival of the surface Si–H bond at 500 °C would be the one that has three oxidized backbonds, i.e., H–Si(O)_3_ [27]. Eventually, surface Si–H disappeared from pSi treated at 600 °C as dehydrogenation takes place, in agreement with a previous observation from annealing a silicon wafer at this temperature [50].

**Figure 2:**
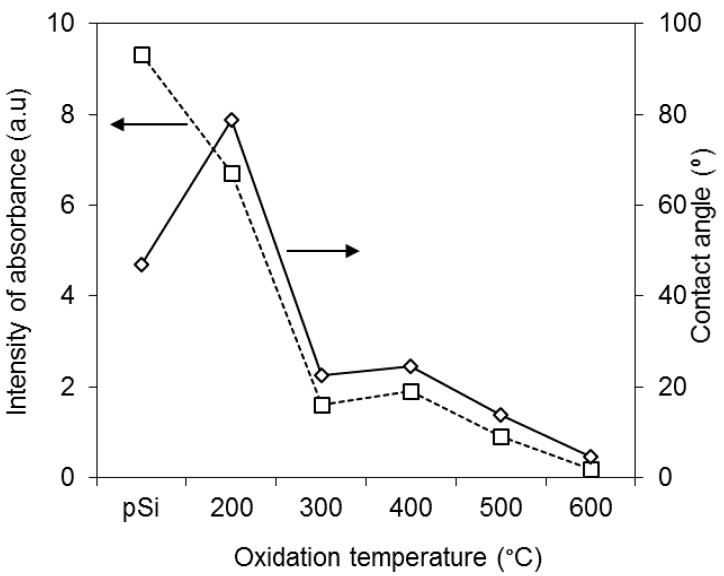
Correlation of the water contact angle measured using the Sessile drop technique (◇) and the integrated absorbance from the Si–Si bending and Si–H_2_ wagging modes at 615–660 cm^−1^ (□) for pSi samples oxidized at 200–600 °C in comparison with unoxidized pSi.

Furthermore, there is a strong correlation between pSi surface chemistry and the water contact angle, as shown in Figure 2 where both the integrated IR absorbance and contact angle are plotted against pSi oxidation temperature.

**Figure 3:**
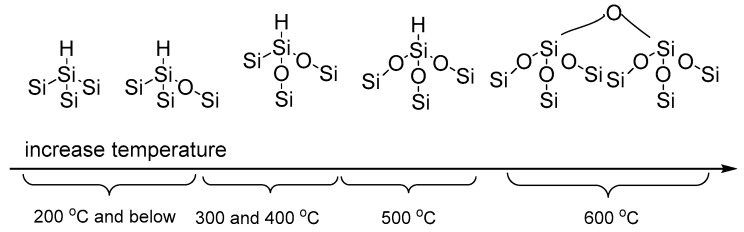
Schematic of pSi surface chemistry (only monohydride is depicted here) during thermal oxidation at elevated temperature.

In summary, the thermal oxidation of pSi particles in this study follows the backbond oxidation phenomenon that has previously been observed with the oxidation of hydride passive silicon wafers [27,47,51,52]. The infrared spectra of pSi samples treated below 400 °C indicate that the Si–Si backbonds of the surface hydrides were only partially oxidized. The Si–Si backbonds were fully oxidized at 500 °C, while at 600 °C, all the surface hydrides were diminished as dehydrogenation occurred, and the bulk Si–Si bonds were also oxidized, as shown schematically in Figure 3.

### 3.2. Dissolution of Unoxidized and Thermally Oxidised pSi

**Figure 4:**
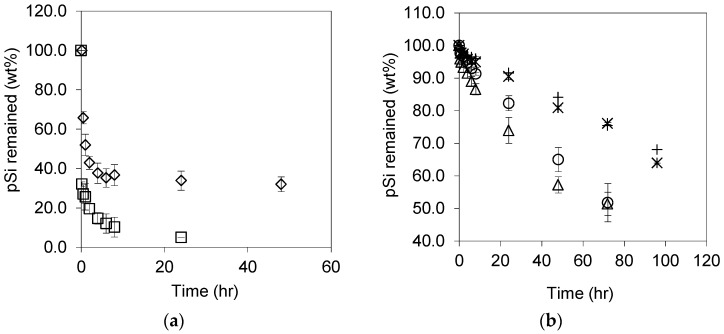
(**a**) Dissolution profiles of the unoxidized pSi (□) and oxidized at 200 °C pSi (◇), and (**b**) samples oxidized at 300 (△), 400 (⚪), 500 (×), and 600 °C (+) (± s.d., *n* = 2).

Dissolution profiles in Tris buffer solution at pH 7.2 for pSi particles of different levels of oxidation are presented in Figure 4. Unoxidized/native pSi particles rapidly dissolve (see Figure 4a), with approximately 52% *w/w* dissolved before the first sampling time point at 5 min. In comparison, OXpSi-200 °C dissolved 37% of its initial mass in the first 10 min. The dissolution was accompanied by bubbling hydrogen gas due to the hydrolysis of hydrogen passivated surfaces [31,34]. The fast dissolution of the unoxidized pSi continued up to the first hour-time point when about 90% of the initial mass was lost while the remaining 10% pSi particles dissolved markedly slower over 7 h; this was likely due to oxidation that took place concurrently along with dissolution, which resulted in the formation of Si–O bonds which are less reactive than Si–Si bonds.

Dissolution profiles for pSi particles oxidized at temperatures >300 °C are shown in Figure 4b and are different from the unoxidized and OXpSi-200 samples. The more highly oxidized pSi samples dissolved with an almost linear reduction in pSi mass observed over time. The dissolution profiles of pSi particles oxidized at 300 and 400 °C were very similar, with approximately 65% mass reduction after 72 h. In contrast, for pSi particles oxidized at higher oxidation temperatures (500 and 600 °C) a reduction in the dissolution rate was observed, i.e., ~40% pSi remaining after 96 h. This is attributed to differences in the relative reactivity of the pSi species present. Indeed, the distinct difference between the dissolution behavior of the pSi oxidized at 200 versus 300 °C suggests a transition in the relative fraction of reactive to passive Si species upon thermal oxidation, in agreement with the previous observations [47].

**Figure 5:**
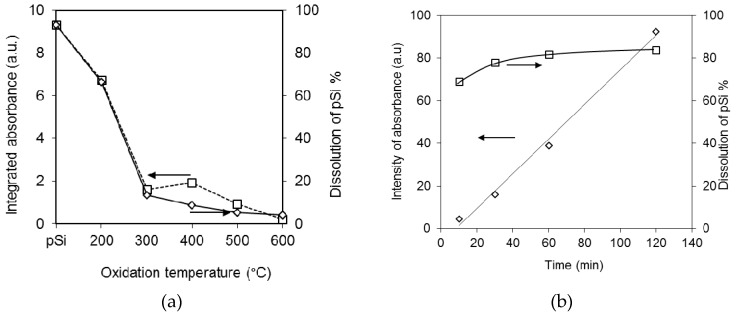
Correlation of pSi surface chemistry as determined with FTIR and dissolution behavior. (**a**) Influence of pSi thermal oxidation temperature on the Si–Si stretching modes (615 cm^−1^) and Si–H_2_ wagging modes (660 cm^−1^) (□) and dissolution of the pSi after 8 h (◇) and (**b**) correlating the integrated absorbance due to Si–O symmetric/asymmetric stretching modes (1080 cm^−1^) (◇) with the % pSi dissolution (□) as a function of dissolution time.

In order to further elucidate the influence of pSi oxidation on the dissolution rate, a parallel experiment was set up, where, at specified time points the dissolution was terminated, and the residual pSi particles were collected and examined with FTIR. The infrared spectra (not shown), indicated that once in contact with the aqueous medium, the pSi simultaneously undergoes both dissolution and oxidation. The proposed mechanism for pSi oxidation in water is oxygen attack of the Si–Si backbond, analogous to pSi oxidation in air [53], where the rate limiting step is oxygen diffusion within the Si–Si lattice [44]. From Figure 5a, the observed decrease in absorbance due to Si–Si stretching modes (at 615 cm^-1^) and Si–H_2_ wagging modes for pSi samples thermally oxidized at increased temperatures correlates well with the extent (%) of pSi dissolution observed after 8 h. This highlights the critical dependence of pSi dissolution on the availability of unoxidized surface hydride species. Similarly, in Figure 5b, a linear (*R*^2^ = 0.99) relationship was observed between the integrated absorbance due to Si–O stretching and dissolution time. This indicates that oxidation of the pSi particles in the dissolution media follows zero order kinetics. Given that ~80% pSi dissolution was observed in the first 30 min, i.e., when the extent of pSi oxidation remains relatively low, the pSi dissolution was not significantly affected by the concurrent oxidation occurring.

### 3.3. Influence of Simulated GI Fluids on pSi Dissolution

**Figure 6:**
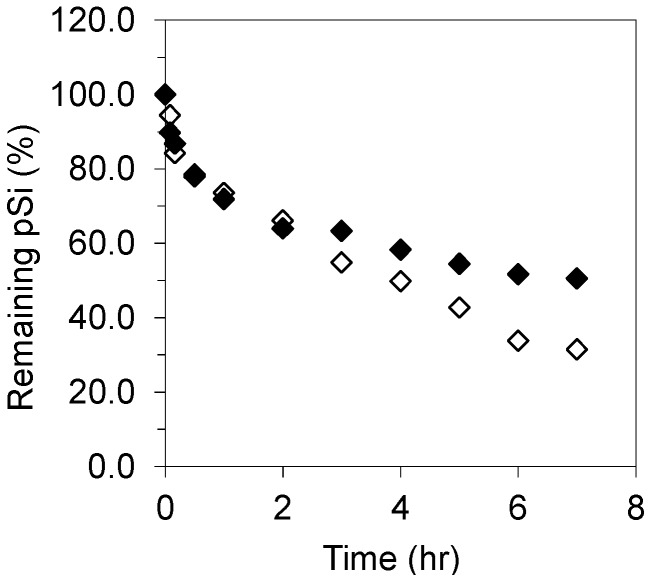
Unoxidised pSi particle dissolution in simulated gastric fluids (SGF) in the presence of pepsin (◆) and absence of pepsin (◇).

Dissolution kinetics of unoxidized pSi microparticles in simulated gastric fluid (SGF) with/without pepsin are given in Figure 6. The dissolution was independent of pepsin addition during the first two hours when 40 wt % of pSi dissolved, but reduced at longer times, i.e., 70 wt % of pSi dissolved at 7 h in the absence of pepsin and 50% dissolved in the presence of pepsin. pSi dissolution in SGF was lower than in Tris buffer solution at pH 7.2, which was expected as the reaction was driven by the nucleophilic attack mechanism of pSi dissolution. The reduced dissolution in the presence of pepsin is considered to be a result of the pepsin protein adsorption on pSi surfaces. Pepsin, a proteolysis enzyme, has a molecular weight ~35 kDa and pI = 1.0 [54]; thus, it is neutral in acidic SGF medium. Moreover, the pSi particles undergo oxidation while in an aqueous medium, i.e., they have surface chemistry akin to hydrophilic silica, possessing a small positive charge at pH 1.0. The pepsin molecule has an estimated hydrodynamic radius of 1.8 nm [55] and, therefore, likely to penetrate the pSi mesoporous network (pore *φ* ≈ 10 nm) in addition to adsorbing at the surface. It has previously been reported that proteins such as albumin, papain, and insulin are able to interact with pSi, as well as being able to penetrate into the porous network [56,57,58].

**Figure 7:**
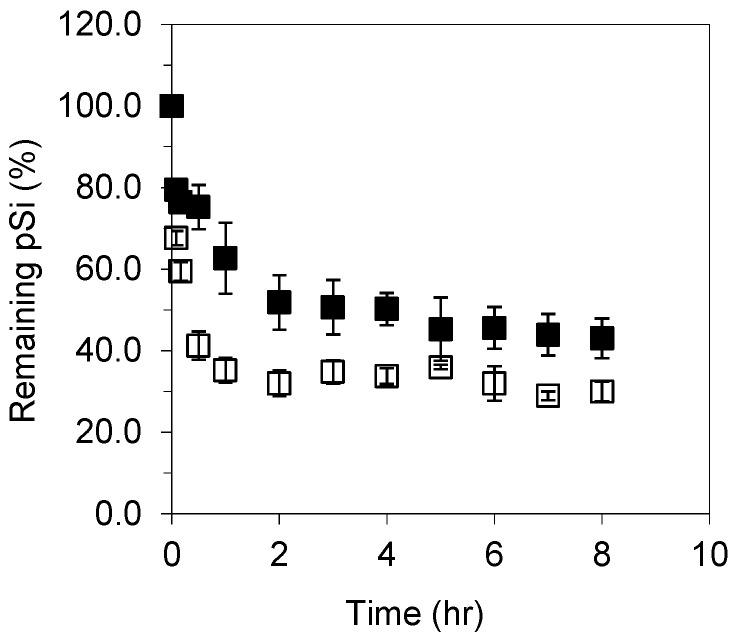
pSi dissolution in simulated intestinal fluids (SIF) in the presence (■) and absence (□) of pancreatin (% ± s.d., *n* = 2).

Dissolution kinetics of unoxidized pSi microparticles in simulated intestinal fluid (SIF) with/without pancreatin are given in Figure 7. In the absence of pancreatin, i.e., phosphate buffer solution (0.05 M) at pH 6.8, pSi exhibited fast dissolution, which accounts for 64 wt % of its initial mass, compared to 90 wt % in Tris buffer solution at pH 7.2 in the first one hour. The difference between pSi dissolution in these two buffer solutions is attributed to their specific components since their pH values were similar. The primary amine moiety in tris(hydroxymethyl)aminomethane (NH_2_C(CH_2_OH)_3_) from Tris buffer might act as a catalyst to hydrolyze surface hydrides of pSi, such catalytic effects of organic amines on pSi hydrolysis were observed previously [34].

In the presence of pancreatin, fast dissolution was observed in the first 2 h, with ~50% of the initial mass dissolved; this was lower than that without pancreatin. Pancreatin is a mixture of the digestive enzymes including trypsin (MW = 23.3 kDa, pI =10.1–10.5), amylase (MW = 50–55 kDa, pI = ~ 6) and lipase (MW = 46 kDa, pI = 7.4) [59]. At neutral pH, trypsin is positively charged, therefore it can undergo electrostatic interactions with the negatively charged unoxidised pSi surface. In contrast, the amylase and lipase molecules are neutral, therefore will only be able to adsorb via weak interactions such as van der Waals forces.

## 4. Discussion

pSi dissolution (as described in Equations (1) and (2)) occurs via a nucleophilic substitution mechanism. Given that the dissolution media volume was far in excess of the sample mass, it can be assumed that pSi dissolution follows pseudo-first order kinetics. However, this was shown not to be the case since a plot of semi-log pSi % remaining versus time data was not linear (data not shown), and more complex kinetics are apparent. The pSi surface is comprised of monohydrides, dihydrides, and trihydrides, each with highly varied activities in aqueous solution. As reported in the literature, the reaction of a vertical dihydride site is five times faster than a terrace monohydride, but two times slower than a kink site (a distorted monohydride) [28]. In addition, dangling bonds might be formed from breaking Si crystalline lattices due to grinding/milling processes. Dangling bonds possess a lower activation barrier for reaction due to lone pair electrons, which are susceptible to water attack [60]. It is assumed that each of the surface species reacted with water at different rates. 

The presence of various reaction sites and the parallel oxidation processes result in more complex dissolution kinetics. The total silicic acid is better described as a sum of silicic acid from each surface species reacted with water. Thus, for the reaction of a surface species, with respect to silicic acid,

(3) $$C_{t}=A_{\infty }\left(1-e^{k_{a}t}\right)$$

where *k* is the rate constant *C_t_*, *A_∞_* and *B_∞_* are concentration of silicic acid at time, *t* and ∞.

(4) $$C_{t}=A_{\infty }\left(1-e^{k_{a}t}\right)+B_{\infty }\left(1-e^{k_{b}t}\right)$$

For the total silicic acid from sample;

(5) $$C_{\infty }=A_{\infty }+B_{\infty }$$

Thus, Equation (3) can be combined with Equation (5) in a natural logarithmic form

(6) $$\begin{array}{ll} ln\left(\frac{C_{\infty }-C_{t}}{C_{\infty }}\right) & =ln\left[\frac{A_{\infty }}{\left(A_{\infty }+B_{\infty }\right)}\cdot e^{-k_{f}t}+\frac{A_{\infty }}{\left(A_{\infty }+B_{\infty }\right)}\cdot e^{-k_{s}t}\right]\\ & =ln\left(P_{f}\cdot e^{-k_{f}t}+P_{S}\cdot e^{-k_{s}t}\right) \end{array}$$

where *P* indicates the relative fraction of fast (*P_f_*) and slow (*P_s_*) dissolving species, and *P_f_* + *P_s_* =1.

This multi-exponential (or the sum of exponential model) approach was used by Truesdale et al. to describe biosilica dissolution data [61]. The model fit agreed well with the postulation that the total surface of biosilica arose from two separate fractions, fast-dissolving and slow-dissolving. However, in the current study, the best linear regression from fitting a simple exponential model to the full dissolution data of unoxidized and oxidized at 200 °C suggested the pSi surface consisted of three separate fractions, fast-, medium- and slow-dissolving surface species (reactive sites). The parameters obtained from this data fit are summarized in Table 2. Therefore:

(7) $$ln\left(\frac{C_{\infty }-C_{t}}{C_{\infty }}\right)=ln\left(P_{f}\cdot e^{-k_{f}t}+P_{m}\cdot e^{-k_{m}t}+P_{S}\cdot e^{-k_{s}t}\right)$$

where *P_f_* + *P_m_* + *P_s_* = 1. The left term in Equation (7) is equivalent to the ratio of the remaining pSi on the assumptions that (a) pSi will be completely converted to silicic acid at infinity time, and (b) there is no side reaction nor reverse reaction, and silicic acid is the only product. This is rational according to the reaction mechanism described earlier since all surface hydrides react with water to form silicic acid, a new layer of hydride would be reconstructed and ready to next cycle of reactions.

As shown in Table 2, the fast fraction of dissolutions from both the pSi samples consists of only two data points (from time zero to 10 min) when pSi dissolved extremely fast and accounted about 69% and 24% of the samples’ dissolution (estimated from the intercept of linear regression), respectively. The dissolved sample mass and rate constant from pSi are three times higher than from OXpSi-200. The fractions of the medium and slow dissolution phases from the unoxidized pSi are equivalent, i.e., 15.5% and these two fractions from pSi oxidized at 200 °C were about two times higher than the fast one. In contrast, the remaining 31% of pSi dissolved slowly (slower than dissolution of the second fraction from 200 °C). These regions of slow dissolution were likely due to oxidation of pSi that has taken place concurrently during dissolution.

The third fraction from OXpSi-200 accounts for 44% of the sample mass, which dissolved with a rate constant similar to those of pSi oxidized at 300 and 400 °C. The difference between dissolution observed from OXpSi-200 and pSi clearly indicated changes in their physicochemical properties. First, oxidation has eliminated the highly reactive dangling bonds and trihydrides (only one Si–Si backbond attached), which contributes to the initial fast dissolution phase. This is in agreement with the observation from the study of heat annealing of a silicon wafer, from which the number of dangling bonds decreased significantly at 200 °C [62], while the surface hydrides from a hydrogen passivated silicon wafer were showed to be relatively stable [63]. Secondly, ~42% of OXpSi-200 has a similar oxidation state to OXpSi-300 and OXpSi-400, confirming the impact of temperature to pSi oxidation.

**Figure 8:**
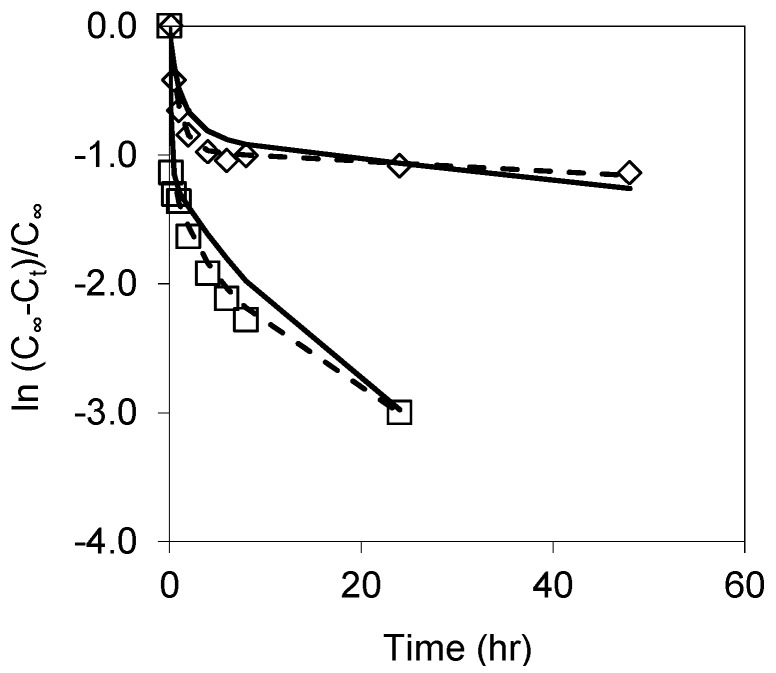
Fitting of simulated data (solid lines) obtained with the sum of exponentials model (Equation (7)) and with adjusted variables (dash lines) over the plots of ln(C_∞_−C*_t_*)/C_∞_ versus time from dissolutions of pSi (□) and OXpSi-200 (◇).

The fits from the simulated data calculated from Equation (7) using the variables given in Table 2 are presented in Figure 8. For both the data sets, the simulation data did not fit precisely, i.e., the predicted fraction of slow dissolution from pSi and the medium dissolution phase from OXpSi-200 deviated from the observed experimental data. A better fit with adjusted variables for pSi (dash line) was achieved if *k*_m_ = 0.4 h^−1^, which is double the original value. Similarly, better fitting for the OXpSi-200 data (dash line), was achieved with *k*_m_ = 0.92 h^−1^ and the fractions of medium and slow reactions, *P*_m_ = 0.28 and *P*_s_ = 0.38. For pSi dissolution, including the 24 h point was the major attribute to the underestimated rate constant (N.B. without 24 h point, *k*_s_ = 0.107 h^−1^). After 8 h, ~90% of pSi dissolved, generating a silicic acid concentration of ~3.2 mM, i.e., just above its saturated concentration (2 mM at 40 °C ). The consequent polymerization of silicic acid could decrease the rate of pSi dissolution. For OXpSi-200, the fraction of slow reaction included ~30% of the total sample mass of undissolved pSi, which might cause overestimated dissolution.

**Figure 9:**
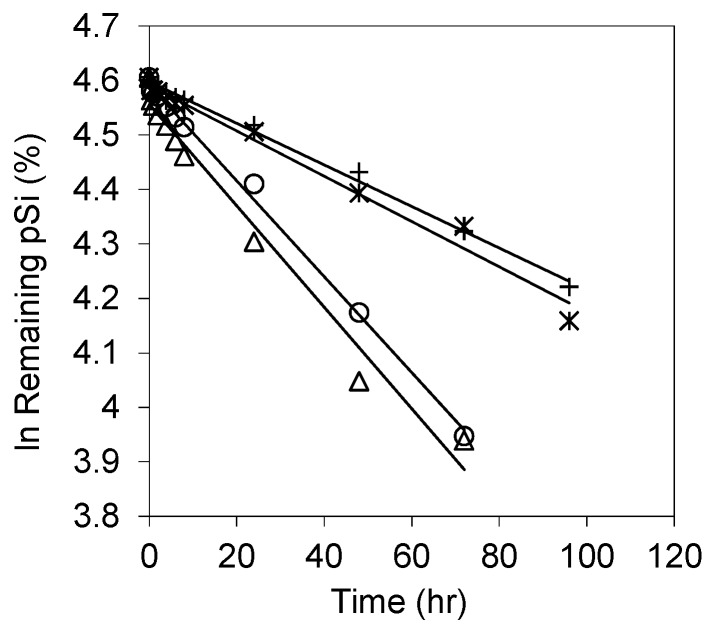
Plots of semi-log the remaining pSi % versus time and least square fittings to the data of dissolution of pSi samples oxidized at above 300 °C. The parameters are given in Table 3.

Dissolution kinetics of the pSi samples oxidized at ≥300 °C are presented in Figure 9, with all data sets exhibiting good linearity from the semi-log concentration-time plots, confirming a simple exponential model (fit and rate constant are shown in Table 3). This suggests that the thermally oxidized (>300 °C) pSi possesses a more homogeneous surface, i.e., a uniformity of surface species or reaction sites, as confirmed by FTIR. 

**Figure 10:**
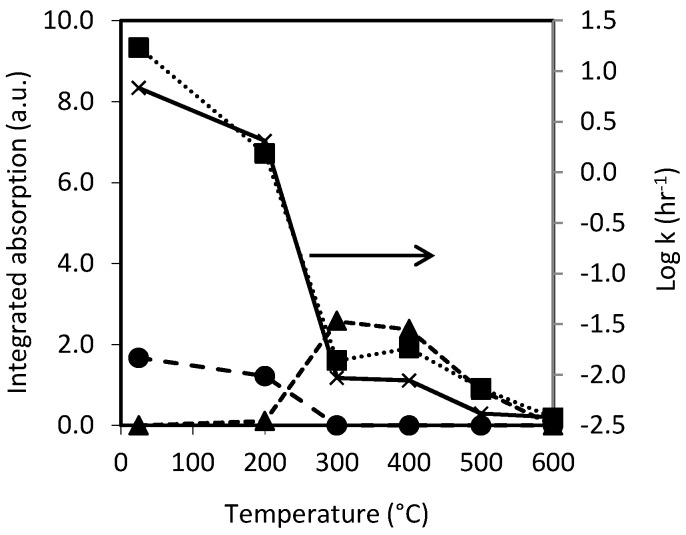
A comparison of rate constants for dissolution and the integrated IR absorption intensity for pSi samples that were oxidized at various temperatures. The temperature for native pSi was set at 25 °C as a symbolic temperature. Absorption at 615 cm^−1^ due to Si–Si (■, dot line), at 2025–2150 cm^−1^ due to Si–Hx (●, dash line) and at 2150–2325 cm^−1^ due to OSi–Hx (▲, short dash line), and rate constant, *k*, (corresponds to the right Y axis) (×, solid line). The values of rate constant for pSi and OXpSi-200 were from the fast dissolution phase.

A positive correlation between the dissolution rate constant and surface properties (IR absorption) of pSi was demonstrated in Figure 10. The rate constant of dissolution decreased when oxidation temperature increased, and this is consistent with decreasing intensities of Si–Hx and Si–Si. The IR absorption band of Si–Hx disappeared at 300 °C while the intensity of Si–Si absorption decreased dramatically from 200 to 300 °C. Furthermore, the IR absorbances due to the hydrides whose backbonds were oxidized (OSi–Hx) increased sharply. From 300 °C, this band decreased gradually along with the increase of temperature and eventually disappeared at 600 °C. From our surface characterization studies we know that at 200 °C, there are surface hydrides in their intermediate oxidation states, i.e., Si atoms bonded to one, two, or three oxygen atoms. At 300 °C, only the oxidized hydrides remained. Breaking Si–O–Si bonds, in fact, share the same mechanism as breaking Si–Si bonds in pSi dissolution, i.e., a nucleophilic substitute with OH^-^ ions [64,65].

Our dissolution data showed that the fast dissolution fraction of pSi oxidized at 200 °C, which predominantly is due to surface hydrides (Si–SiHx) was ~1000 times faster than the dissolution of pSi oxidized at 300 °C, which consists of oxidized hydrides (Si–O–SiHx). Dissolution of pSi samples oxidised at ≥300 °C were similar, because all the surface hydrides were in various intermediate oxidation states, to release a Si atom (in silicic acid form), i.e., breaking not only Si–Si bonds but also breaking the newly forming Si–O–Si backbonds which becomes a rate-limiting step for the pSi dissolution. Also, it is worthy of note that the formation of Si–O–Si bonds due to thermal oxidation can cause geometric changes of the pSi lattice resulting in both negative and positive impacts to dissolution. In the former case, expanded crystalline lattice results in reduced surface area and pore volume [66]; while in the latter, strain and deformation of lattice would be in favor of dissolution [67].

## 5. Conclusions

The dissolution kinetics of pSi microparticles can be extensively controlled by thermal oxidation (200–600 °C). Unoxidized pSi and OXpSi-200 dissolved fastest at pH 7.2, which was attributed to the presence of the different surface hydride groups which exhibit different reaction rates. Thermal oxidation has a significant impact on the pSi dissolution mechanism, and pSi oxidized at above 300 °C following pseudo-first order dissolved kinetics, this is due to the rate-limiting step of breaking oxidized backbonds (Si–O–Si). We have successfully developed an exponential kinetic model to describe pSi dissolution, which accounts for the influence of Si speciation. Finally, we investigated the influence of simulated gastric and intestinal fluids on unoxidized pSi dissolution, and showed the presence of GI proteins reduces pSi dissolution; this is presumed do to surface adsorption to pSi and protection against water attack of the Si surface species. These findings provide insight into key considerations for future formulation optimization of specific pSi based biomaterials, particularly for their use in oral drug delivery.

## Figures and Tables

**Table 1:** Surface area and pores volume/size of pSi samples.

Oxidation Temperature (°C)	Label	BET-SSA (m^2^/g)	Mesopore Volume (mL/g)	Mesopore Diameter (nm)
unoxidized pSi	pSi	325.7	0.77	10.1
200	OXpSi-200	312.5	0.74	10.1
300	OXpSi-300	-	-	-
400	OXpSi-400	247.7	0.61	9.6
500	OXpSi-500	-	-	-
600	OXpSi-600	243.8	0.48	9.3

**Table 2:** Parameters from fitting multi-exponential equation to dissolution of unoxised and 200 °C oxidized pSi particles.

Sample	Unoxidized pSi			OXpSi-200		
	Fast	Medium	Slow	Fast	Medium	Slow
Rate constant *k* (h^−1^)	6.82	0.19	0.0475	2.05	0.463	0.00824
Fraction (P)	0.690	0.155	0.155	0.340	0.240	0.420
Time points	0–10 min	10–240 min	360 min–24 h	0–10 min	10–60 min	360 min–48 h
*R* ^2^	1.0 *	0.9673	0.9944	1.0 *	0.9951	0.9217

* Denotes that only two data points are included.

**Table 3:** The parameters obtained from applying least square to plot of logarithmic % remaining sample versus time from dissolutions of pSi particles oxidized at ≥300 °C.

Sample	Rate Constant (×10^−3^ h^−1^)	*t*_½_ (h)	*R* ^2^
OXpSi-300	9.300	74.5	0.9778
OXpSi-400	8.797	78.8	0.9967
OXpSi-500	4.153	166.9	0.9823
OXpSi-600	3.811	181.8	0.9951

## References

[1] Canham L.T. (1995). Bioactive silicon structure fabrication through nanoetching techniques. Adv. Mater..

[2] Korhonen E., Rönkkö S., Hillebrand S., Riikonen J., Xu W., Järvinen K., Lehto V.-P., Kauppinen A. (2016). Cytotoxicity assessment of porous silicon microparticles for ocular drug delivery. Eur. J. Pharm. Biopharm..

[3] Tzur-Balter A., Shatsberg Z., Beckerman M., Segal E., Artzi N. (2015). Mechanism of erosion of nanostructured porous silicon drug carriers in neoplastic tissues. Nat. Commun..

[4] Maniya N.H., Patel S.R., Murthy Z.V.P. (2015). Development and in vitro evaluation of acyclovir delivery system using nanostructured porous silicon carriers. Chem. Eng. Res. Des..

[5] Wang F., Hui H., Barnes T.J., Barnett C., Prestidge C.A. (2009). Oxidized Mesoporous Silicon Microparticles for Improved Oral Delivery of Poorly Soluble Drugs. Mol. Pharm..

[6] Qi S.C., Zhang P., Ma M., Yao M., Wu J., Mäkilä E., Salonen J., Ruskoaho H., Xu Y., Santos H.A. (2019). Cellular Internalization-Induced Aggregation of Porous Silicon Nanoparticles for Ultrasound Imaging and Protein-Mediated Protection of Stem Cells. Small.

[7] Galushka V.V., Belobrovaya O.Y., Bratashov D.N., Kondrateva O.Y., Polyanskaya V.P., Sidorov V.I., Yagudin I.T., Terin D.V. (2018). Gamma-Radiation Monitoring of Luminescent Porous Silicon for Tumor Imaging. Bionanoscience.

[8] Yang Y., Su P., Tang Y. (2018). Stimuli-Responsive Lanthanide-Based Smart Luminescent Materials for Optical Encoding and Bio-applications. ChemNanoMat.

[9] Kumeria T., McInnes S.J.P., Maher S., Santos A. (2017). Porous silicon for drug delivery applications and theranostics: Recent advances, critical review and perspectives. Expert Opin. Drug Deliv..

[10] Barnes T.J., Prestidge C.A. (2015). Recent advances in porous silicon-based therapeutic delivery. Ther. Deliv..

[11] Jakobsson U., Makila E., Airaksinen A.J., Alanen O., Etile A., Koster U., Ranjan S., Salonen J., Santos H.A., Helariutta K. (2019). Porous Silicon as a Platform for Radiation Theranostics Together with a Novel RIB-Based Radiolanthanoid. Contrast Media Mol. Imaging.

[12] Wang C.-F., Sarparanta M.P., Mäkilä E.M., Hyvönen M.L.K., Laakkonen P.M., Salonen J.J., Hirvonen J.T., Airaksinen A.J., Santos H.A. (2015). Multifunctional porous silicon nanoparticles for cancer theranostics. Biomaterials.

[13] Prestidge C.A., Barnes T.J., Santos H.A. (2014). 15-Nanoporous silicon to enhance drug solubility. Porous Silicon for Biomedical Applications.

[14] Merazga S., Cheriet A., M’Hammedi K., Mefoued A., Gabouze N. (2019). Investigation of porous silicon thin films for electrochemical hydrogen storage. Int. J. Hydrogen Energy.

[15] Tang C.-H., Li W.-J., Hung C.-H., Hsiao P.-H., Chen C.-Y. (2017). Highly Porous Silicon Nanowires Made with Solvent-Mediated Wet Chemical Etching and Their Thermoelectric Applications. ChemistrySelect.

[16] Xia B., Zhang W., Shi J., Li J., Chen Z., Zhang Q. (2019). NIR light-triggered gelling in situ of porous silicon nanoparticles/PEGDA hybrid hydrogels for localized combinatorial therapy of cancer cells. J. Appl. Polym. Sci..

[17] Kurdyukov D.A., Eurov D.A., Shmakov S.V., Kirilenko D.A., Kukushkina J.A., Smirnov A.N., Yagovkina M.A., Klimenko V.V., Koniakhin S.V., Golubev V.G. (2019). Fabrication of doxorubicin-loaded monodisperse spherical micro-mesoporous silicon particles for enhanced inhibition of cancer cell proliferation. Microporous Mesoporous Mater..

[18] Kang R.H., Lee S.H., Kang S., Kang J., Hur J.K., Kim D. (2019). Systematic Degradation Rate Analysis of Surface-Functionalized Porous Silicon Nanoparticles. Materials.

[19] Li W., Liu Z.H., Fontana F., Ding Y.P., Liu D.F., Hirvonen J.T., Santos H.A. (2018). Tailoring Porous Silicon for Biomedical Applications: From Drug Delivery to Cancer Immunotherapy. Adv. Mater..

[20] Seo H., Kim D., Ahn H.S., Hwang S., Luu Q.S., Kim J., Lee S., Lee Y. (2018). Efficient Conversion Method of Bulk Silicon Powders into Porous Silicon Nanoparticles. Bull. Korean Chem. Soc..

[21] Jarvis K.L., Barnes T.J., Prestidge C.A. (2012). Surface chemistry of porous silicon and implications for drug encapsulation and delivery applications. Adv. Colloid Interface Sci..

[22] Jarvis K.L., Barnes T.J., Prestidge C.A. (2011). Surface chemical modification to control molecular interactions with porous silicon. J. Colloid Interface Sci..

[23] Jarvis K.L., Barnes T.J., Prestidge C.A. (2010). Thermal Oxidation for Controlling Protein Interactions with Porous Silicon. Langmuir.

[24] Mäkilä E., Bimbo L.M., Kaasalainen M., Herranz B., Airaksinen A.J., Heinonen M., Kukk E., Hirvonen J., Santos H.A., Salonen J. (2012). Amine Modification of Thermally Carbonized Porous Silicon with Silane Coupling Chemistry. Langmuir.

[25] Britcher L., Barnes T.J., Griesser H.J., Prestidge C.A. (2008). PEGylation of porous silicon using click chemistry. Langmuir.

[26] Zhang D.X., Yoshikawa C., Welch N.G., Pasic P., Thissen H., Voelcker N.H. (2019). Spatially Controlled Surface Modification of Porous Silicon for Sustained Drug Delivery Applications. Sci. Rep..

[27] Ogata Y.H., Kato F., Tsuboi T., Sakka T. (1998). Changes in the environment of hydrogen in porous silicon with thermal Annealing. J. Electrochem. Soc..

[28] Hines M.A., Chabal Y.J., Harris T.D., Harris A.L. (1994). Measuring the structure of etched silicon surfaces with Raman spectroscopy. J. Chem. Phys..

[29] Allongue P., Brune H., Gerischer H. (1992). In situ STM observations of the etching of n-Si(111) in NaOH solutions. Surf. Sci..

[30] Campbell S.A., Schiffrin D.J., Tufton P.J. (1993). Chemical and electrochemical anisotropic dissolution of silicon in ethylenediamine + pyrocatechol + water media. J. Electroanal. Chem..

[31] Steinem C., Janshoff A., Lin V.S.Y., Volcker N.H., Ghadiri R.M. (2004). DNA hybridization-enhanced porous silicon corrosion: Mechanistic investigations and prospect for optical interferometric biosensing. Tetrahedron.

[32] Zhu G., Liu J.-T., Wang Y., Zhang D., Guo Y., Tasciotti E., Hu Z., Liu X. (2016). In Situ Reductive Synthesis of Structural Supported Gold Nanorods in Porous Silicon Particles for Multifunctional Nanovectors. ACS Appl. Mater. Interfaces.

[33] Bateman J.E., Eagling R.D., Horrocks B.R., Houlton A., Worrall D.R. (1997). Role for organic molecules in the oxidation of porous silicon. Chem. Commun..

[34] Xu D., Sun L., Li H., Zhang L., Guo G., Zhao X., Gui L. (2003). Hydrolysis and silanization of the hydrosilicon surface of freshly prepared porous silicon by an amine catalytic reaction. New J. Chem..

[35] Floch J., Blain S., Birot D., Treguer P. (1998). In situ determination of silicic acid in sea water based on FIA and colorimetric dual-wavelength measurements. Anal. Chim. Acta.

[36] Anderson S.H.C., Elliott H., Wallis D.J., Canham L.T., Powell J.J. (2003). Dissolution of different forms of partially porous silicon wafers under simulated physiological conditions. Phys. Status Solidi.

[37] Manchon A., Alkhraisat M.H., Rueda-Rodriguez C., Pintado C., Prados-Frutos J.C., Torres J., Cabarcos E.L. (2018). Silicon bioceramic loaded with vancomycin stimulates bone tissue regeneration. J. Biomed. Mater. Res. Part B Appl. Biomater..

[38] Low S.P., Voelcker N.H., Canham L.T., Williams K.A. (2009). The biocompatibility of porous silicon in tissues of the eye. Biomaterials.

[39] Bowditch A.P., Waters K., Gale H., Rice P., Scott E.A.M., Canham L.T., Reeves C.L., Loni A., Cox T.I. (1999). In-vivo assessment of tissue compatibility and calcification of bulk and porous silicon. Mater. Res. Soc. Symp. Proc..

[40] Wang F., Timothy J.B., Clive A.P. (2013). Celecoxib confinement within mesoporous silicon for enhanced oral bioavailability. Mesoporous Biomater..

[41] Kato Y., Ito T., Hirak A. (1988). Initial oxidation process of anodized porous silicon with hydrogen atoms chemisorbed on the inner surface Japan. J. Appl. Phys..

[42] Ogata Y., Niki H., Sakka T., Iwasaki M. (1995). Oxidation of porous silicon under water vapor environment. J. Electrochem. Soc..

[43] Bateman J.E., Horrocks B.R., Houlton A. (1997). Reactions of water and methanol at hydrogen-terminated silicon surfaces studied by transmission FTIR. J. Chem. Soc. Faraday Trans..

[44] Haiss W., Raisch P., Schirin D.J., Bitsch L., Nichols R.J. (2002). An FTIR study of the surface chemistry of the dynamic Si(100) surface during etching in alkaline solution. Faraday Discuss.

[45] Chabal Y.J., Raghavachari K. (1985). New ordered structure for the H-Saturated Si(100) surface: The (3x1) Phase. Phys. Rev. Lett..

[46] Bellet D., Dolino G., Ligeon M., Blanc P., Krisch M. (1992). Studies of coherent and diffuse x-ray scattering by porous silicon. J. Appl.Phys..

[47] Mawhinney D.B., Glass J.A., Yates J.T. (1997). FTIR study of the oxidation of porous silicon. J. Phys. Chem. B.

[48] Kato Y., Ito T., Hiraki A. (1989). Low temperature oxidation of crystalline porous silicon. Appl. Surf. Sci..

[49] Graf D., Grundner M., Schulz R. (1989). Reaction of water with hydrofluoric acid treated silicon(111) and (100) surfaces. J. Vac. Sci.Tech. A.

[50] Stefanov B.B., Gurevich A.B., Weldon M.K., Raghavachari K., Chabal Y.J. (1998). Silicon Epoxide: Unexpected Intermediate during Silicon Oxide Formation. Phys. Rev. Lett..

[51] Ogata Y.H., Tsuboi T., Sakka T., Naito S. (2000). Oxidation of porous silicon in dry and wet environments under mild temperature conditions. J. Porous Mater..

[52] Raghavachari K., Chabal Y.J. (2001). First-Principles quantum chemical investigations of silicon oxidation. Fundamental Aspect of Silicon Oxidation.

[53] Morita M., Ohmi T., Hasegawa E., Kawakami M., Suma K. (1989). Control factor of native oxide growth on silicon in air or in ultra-pure water. Appl. Phys. Lett..

[54] Gole A., Sastry M., Dash C., Rao M. (2000). Encapsulation and biocatalytic activity of the enzyme pepsin in fatty lipid films by selective electrostatic interactions. Chem. Commun..

[55] Uversky V.N., Permyakov A.E.A., Uversky V.N. (2007). Methods in Protein Structure and Stability Analysis: Conformational Stability, Size, Shape and Surface of Protein Molecules. Molecular Anatomy and Physiology of Protein.

[56] Prestidge C.A., Barnes T.J., Mierczynska-Vasilev A., Skinner W., Peddie F., Barnett C. (2007). Loading and release of a model protein from porous silicon powders. Phys. Status Solidi A Appl. Mater. Sci..

[57] Foraker A.B., Walczak R.J., Cohen M.H., Boiarski T.A., Grove C.F., Swaan P.W. (2003). Microfabricated porous silicon particles enhance paracellular delivery of insulin across intestinal Caco-2 cell monolayers. Pharm. Res..

[58] Karlsson L.M., Tengvall P., Lundstrom I., Arwin H. (2003). Penetration and loading of human serum albumin in porous silicon layers with different pore sizes and thicknesses. J. Colloid Interface Sci..

[59] Cozzone P., Pasero L., Marchis-Mouren G. (1970). Characterization of Porcine Pancreatic Isoamylases: Separation and Amino Acid Composition. Biochim. Biophys. Acta.

[60] Bozso F., Avouris P. (1986). Reaction of Si(100) with NH_3_: Rate-limiting steps and reactivity enhancement via electronic excitation. Phys. Rev. Lett..

[61] Truesdale V., Greenwood J., Rendell A. (2005). The rate-equation for biogenic silica dissolution in seawater—New hypotheses. Aquat. Geochem..

[62] Lim P.K., Tam W.K., Yeung L.F., Lam F.M. (2007). Effect of hydrogen on dangling bond in a-Si thin film. J. Phys. Conf. Ser..

[63] Trucks G.W., Raghavachari K., Higashi G.S., Chabal Y.J. (1990). Mechanism of HF etching of silicon surfaces: A theoretical understanding of hydrogen passivation. Phys. Rev. Lett..

[64] O’Connor T.L., Greenberg S.A. (1958). The kinetics for the solution of silica in aqueous solutions. J. Phys. Chem..

[65] Bunker B.C. (1994). Molecular mechanisms for corrosion of silica and silicate glasses. J. Non-Cryst. Solid.

[66] Jarvis K.L., Barnes T.J., Badalyan A., Pendleton P., Prestidge C.A. (2008). Impact of thermal oxidation on the adsorptive properties and structure of porous silicon particles. J. Phys. Chem. C.

[67] Pap A.E., Kordas K., Toth G., Levoska J., Uusimaki A., Vahakangas J., Leppavuori S., George T.F. (2005). Thermal oxidation of porous silicon: Study on structure. Appl. Phys. Lett..

